# Quantitative electron phase imaging with high sensitivity and an unlimited field of view

**DOI:** 10.1038/srep14690

**Published:** 2015-10-01

**Authors:** A. M. Maiden, M. C. Sarahan, M. D. Stagg, S. M. Schramm, M. J. Humphry

**Affiliations:** 1 Dept. Electronic & Electrical Engineering, University of Sheffield, Mappin St, Sheffield, S1 3JD; 2 Phase Focus Ltd, Electric Works, Sheffield Digital Campus, Sheffield S1 2BJ; 3 Gatan Inc, 5794 W. Las Positas Blvd, Pleasanton, CA, 94588.

## Abstract

As it passes through a sample, an electron beam scatters, producing an exit wavefront rich in information. A range of material properties, from electric and magnetic field strengths to specimen thickness, strain maps and mean inner potentials, can be extrapolated from its phase and mapped at the nanoscale. Unfortunately, the phase signal is not straightforward to obtain. It is most commonly measured using off-axis electron holography, but this is experimentally challenging, places constraints on the sample and has a limited field of view. Here we report an alternative method that avoids these limitations and is easily implemented on an unmodified transmission electron microscope (TEM) operating in the familiar selected area diffraction mode. We use ptychography, an imaging technique popular amongst the X-ray microscopy community; recent advances in reconstruction algorithms now reveal its potential as a tool for highly sensitive, quantitative electron phase imaging.

Ptychography was originally suggested as a point-scanning solution to the phase problem^1^,^2^,^3^, but over the past decade has been reimagined as a form of Coherent Diffractive Imaging (CDI)^4^. Ptychographic CDI involves translating a specimen to a series of positions relative to a localised ‘probe’ of illumination and recording a diffraction pattern at each position^5^. Given partial overlap of the areas of the specimen illuminated by the probe and an appropriate model of the formation of the diffraction pattern, iterative algorithms can solve the inverse problem of determining the complex-valued sample transmission function that produced the recorded data^6^,^7^,^8^,^9^,^10^. Electron ptychographic CDI has been implemented at atomic resolution^11^,^12^, but most applications lie at longer lengthscales^13^ and require a phase sensitivity and accuracy that ptychography has yet to demonstrate. An early iteration at nanometre resolution gave only a 0.3 rad accuracy^14^, due to experimental constraints and the limited computational tools available at the time^15^; using new algorithms and a new experimental procedure, the results we present here significantly improve on these images.

Ptychographic CDI is remarkably adaptable. Three-dimensional imaging^16^, reconstruction from noisy data^17^ and a relaxation of sampling constraints^18^ are all possible, partial coherence of several flavours can now be accommodated^19^,^20^,^21^,^22^, and errors in the specimen position grid of up to 45% of the probe diameter^23^,^24^,^25^ can be corrected. The new computational tools that facilitate this flexibility are key components of our implementation here: accurate positioning at the nanoscale and below is difficult and expensive, but positioning errors in the tens of nanometres range were corrected as part of our image reconstruction process; the TEM’s lens instabilities, finite source size and finite bandwidth reduce coherence, as does inelastic scattering within the specimen, yet we were able to computationally compensate for these effects and did not need to use energy filtering in our experiments.

Because we operate in the selected area diffraction mode familiar to most TEM users, we refer to our technique as selected area ptychography, or SAP.

## Results

**Figure 1:**
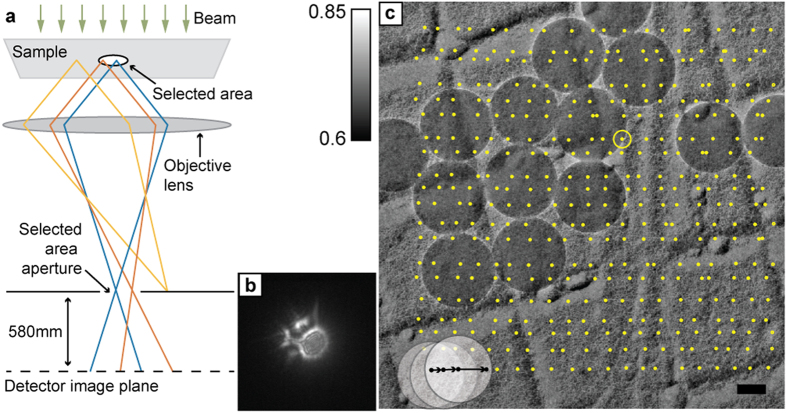
Description of the experiments. The microscope was operated in selected area diffraction mode, illustrated by the ray diagram in (**a**), with a large defocus introduced so that the detector imaged a plane 580 mm beneath the selected area aperture. This resulted in diffraction patterns similar to the example shown in (**b**), where the square-root of the recorded intensity is displayed to provide contrast. Diffraction patterns were recorded over the grid of 20 rows and 20 columns indicated by the dots over the brightfield survey image in (**c**) (square-root of intensity is shown). The first three regions masked by the selected area aperture are highlighted, and the position that resulted in the recorded data shown in (**b**) is circled. Scale bar 100 nm.

Figure 1a illustrates the experimental setup for SAP. A specimen is illuminated by an approximately parallel electron beam. An objective lens collects and focuses scattered electrons to form an image in the plane of the selected area aperture (SAA). The aperture masks the image, allowing through only electrons originating from the small region of the specimen indicated, and thus acts as a ‘virtual’ ptychographic probe in this geometry. The intermediate lenses of the microscope are adjusted so that the detector images a plane 580 mm beneath the SAA, resulting in the disc-like diffraction patterns exemplified by Fig. 1b. These patterns are essentially defocussed selected area images and the data we collect is similar to that used for X-ray near-field ptychography^26^ and for inline holography^27^. Capturing data in the near-field is easier than is the case for far-field diffraction patterns, which have a high dynamic range and an intense central peak that often requires a beamstop.

The brightfield TEM image in Fig. 1c shows our test specimen, a gold-shadowed carbon diffraction grating replica populated by polystyrene spheres 260 nm in diameter. It was chosen to provide phase images with a range of spatial frequencies and to allow quantification of accuracy and sensitivity, as we will see later. The specimen was translated in a raster fashion to sequentially centre the locations indicated by the dots in Fig. 1c under the SAA—the circled position corresponds to the diffraction pattern in Fig. 1b and the discs bottom left show the first three areas isolated by the SAA. One diffraction pattern per specimen position was recorded and together these formed the input to the reconstruction algorithm.

Over the course of data collection a slow drift of the optic axis across the CCD camera was observed—this can be seen clearly in Supplementary Video 1. The same effect was noted previously by Hue *et al.*^14^, who compensated for it prior to image reconstruction via cross-correlation with a reference pattern; we have refined this approach, correcting the drift during image reconstruction as described in the Supplementary Information. This was effective for the chosen test specimen, but slow phase gradients in some samples will cause a real translation of the diffraction pattern that our algorithm may mistakenly remove. Consequently we are investigating changes in the microscope setup and speedups to our data collection process that will eliminate optic axis drift, which we think is caused by slow variations in the intermediate lens currents.

**Figure 2:**
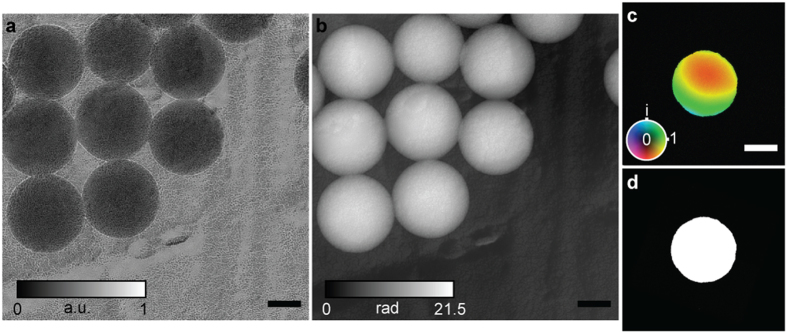
An initial reconstructions. (**a**) Modulus and (**b**) unwrapped phase images of 260 nm diameter polystyrene spheres on a gold-shadowed carbon diffraction grating replica. Scale bars 100 nm. (**c**) A colour wheel plot of the reconstructed selected area aperture: here colour encodes phase variations and brightness amplitude variations. The reconstructed aperture shape is in excellent agreement with (**d**), a brightfield image of the aperture. Scale bar 5 μm. Note: (**c**) has been rotated and cropped to match the scale and orientation of (**d**).

Image reconstructions were carried out using the modified version of the ‘ePIE’ algorithm^8^ detailed in the Supplementary Information (without the improvements detailed there we were not able to produce a sensible result). Amplitude and unwrapped phase images from a first data set are shown in Fig. 2a,b respectively. Note the improved contrast in the modulus image in comparison to Fig. 1c (modulus rather than intensity is displayed in both cases); this is partly a consequence of the removal of inelastic scatter from the diffraction data, which contributes a contrast-reducing background to the brightfield image. Figure 2c shows the image of the SAA, which is also recovered as part of the reconstruction process. The colour-wheel representation highlights phase variations across the SAA—these are not a property of the aperture itself but instead correspond to deviations from a perfect plane wave of the incident electron beam. A 10–90% intensity criterion applied to a line plot across the edge of the SAA reconstruction produced a resolution estimate of 100 nm, and dividing this figure by the objective lens magnification gave an estimated resolution of 2.1 nm in the specimen images. The shape of the SAA reconstruction agrees well with the brightfield image of the aperture shown in Fig. 2d.

**Figure 3:**
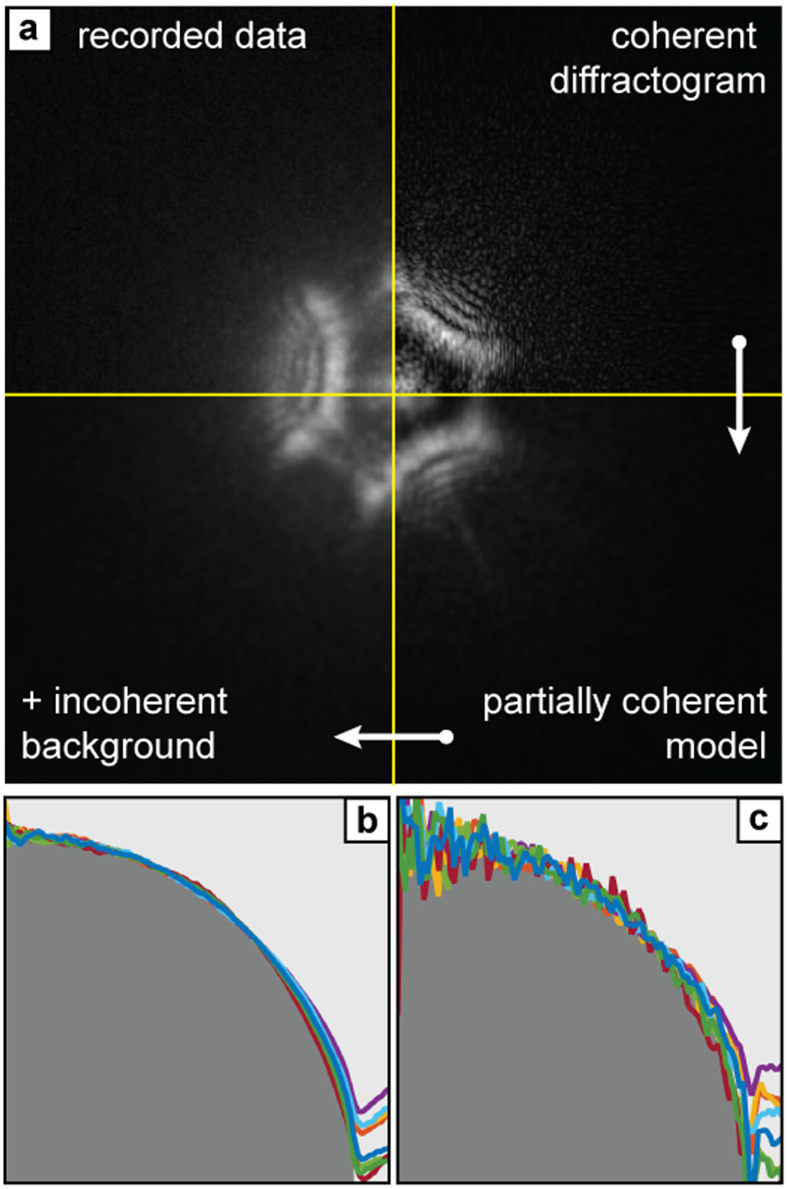
Analysis of the accuracy of the results. (**a**) Using the final reconstructed images of the specimen and the selected area aperture, the forward model of diffraction pattern formation produces excellent agreement with the recorded data. The model consists of three stages, described clockwise from top right: a fully coherent diffraction pattern is simulated; partial spatial coherence is introduced via a convolution; a diffuse incoherent background is added. The polystyrene spheres of the test specimen provide a further means to assess the accuracy of the reconstructions. Each coloured trace in (**b**) plots a radial average over the unwrapped phase image of one of the spheres. The plots closely follow the expected spherical profile (indicated by grey shading) and can be used to measure the mean inner potential of the polystyrene. In (**c**) the log of the modulus image was used to compute a second set of radial averages (shown again by coloured traces), which also have the expected spherical profile; this data can be used to calculate the inelastic mean free path of the polystyrene.

Accurate reconstructions of the specimen and the SAA, when fed into an appropriate forward model, should produce diffraction patterns in close agreement with the measured data. The model in our case is illustrated by Fig. 3a and comprised the following steps. The interaction of the SAA with the specimen image formed by the objective lens was modelled by a multiplication. Propagation of the resulting wavefront to the plane of the CCD used a Fresnel propagator and produced fully coherent diffraction patterns, as exemplified in the top right of Fig. 3a. (Note that the model used in previous versions of electron ptychography stops at this point.) The reduced fringe contrast caused by partial spatial coherence in the illumination was next incorporated by convolving the coherent diffraction pattern with a Gaussian kernel whose width was iteratively refined during the reconstruction^28^ (bottom right of Fig. 3a). Finally, a background function was added, constant in form at each specimen position but of varying weight, to account for an incoherent inelastic background present in the electron wavefront at the plane of the SAA (bottom left of Fig. 3a). This function and the variable weightings were determined by the reconstruction algorithm. The resulting model diffraction pattern shows excellent agreement with the corresponding recorded data (top left of Fig. 3a), and Supplementary Video 2 shows that this is the case at every specimen position.

**Figure 4:**
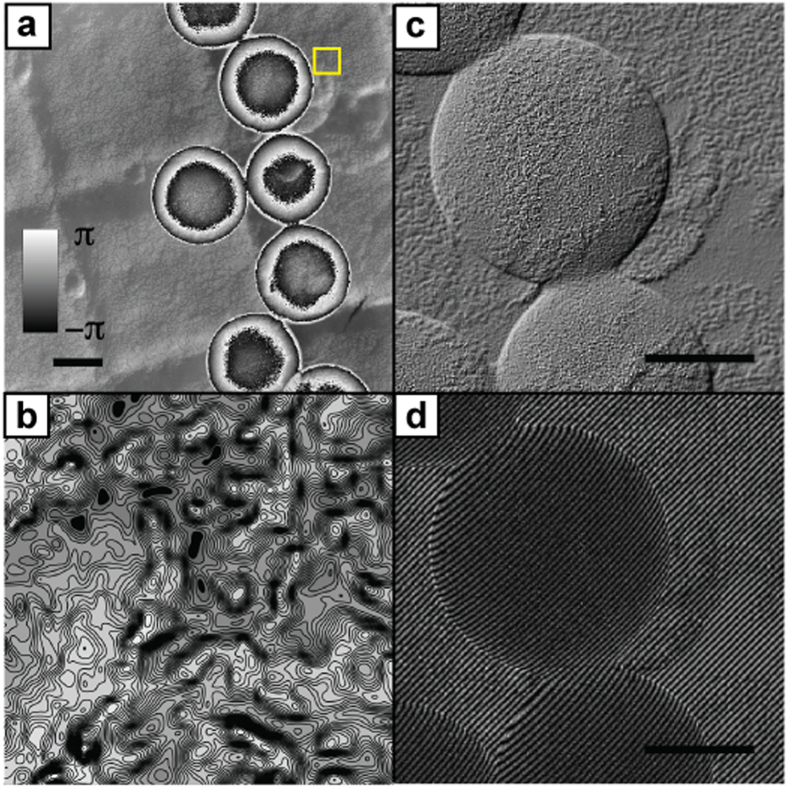
Results from a second experiment, displayed in a range of formats. (**a**) The wrapped phase image. (**b**) A contour plot from the area indicated by the square in (**a**); contours have a spacing of 2π/100 radians. (**c**) A cutout from a differential interference contrast (DIC) image simulated from the data in (**a**), highlighting the accurate reconstruction of the diffraction replica beneath the polystyrene spheres and the texture of the spheres themselves. (**d**) A cutout from an off-axis hologram simulation, again using the data from (**a**). Recording this hologram in practice would not be possible as there is no adjacent vacuum region from which to derive a suitable reference wave. Scale bars 100 nm.

The polystyrene balls populating our test specimen allowed further assessment of phase image accuracy. We extracted radial averages of the unwrapped phase for each of the eight balls in Figure 2b and found best fits to a perfect sphere—Fig. 3b shows the resulting matches. The constant of proportionality derived from the fitting relates directly to the mean inner potential of polystyrene, with radial averages from this and a second set of data (see Fig. 4) giving a value of 7.9 V (standard deviation: 0.64 V), in excellent agreement with the literature^29^. Applying the same fitting process to the log of the modulus image gave the matches shown in Fig. 3c. The fit parameters here were used to calculate an inelastic mean free path for the polystyrene of 125 nm (standard deviation: 21 nm), again agreeing with the literature^30^ and indicating that the inelastic background has been suppressed in the ptychoraphic reconstruction. The accuracy of these figures is primarily determined by the effect of the underlying support film on the radial profiles of the polystyrene spheres, but is subject as well to errors in the manually-chosen sphere centre points and radii, and to noise in the images. Further details of the fitting process can be found in the Supplementary Information.

To demonstrate the sensitivity of our method to small variations in phase, we repeated our experiment on a different area of the same test specimen, producing the wrapped phase image shown in Fig. 4a. Figure 4b shows a contour map of the region highlighted by the square in Fig. 4a—contours are spaced 2π/100 rad apart, but resolve the fine structure of phase variations caused by the gold-shadowing of the carbon replica. Figure 4c provides further evidence of this sensitivity—it shows a cutout from a Diffraction Interference Contrast (DIC) image simulated from the ptychographic reconstruction, which highlights the topology of the grating replica recovered beneath the polystyrene spheres as well as the textured surface of the spheres themselves. Figure 4d shows a similar cutout from a simulated off-axis hologram: it would be impossible to record this hologram in practice, as there is no vacuum region near the sample from which the reference wave could be derived.

A final representation of our results is given in Supplementary Video 3, which shows how measurement of the electron wavefront makes computational refocussing possible. Notice in the video the slight lensing effect of the polystyrene spheres on the carbon substrate imaged through them.

## Discussion

Quantitative imaging of phase in the TEM is predominantly carried out by off-axis holography^13^,^31^, which has realised great success in experienced hands. Holography in an off-axis geometry has the advantage of being a one-shot method, requiring only a single careful exposure, and the algorithms used to extract the phase image are quick and simple. However, it is not readily accessible to most TEM users: the instrument must be equipped with an electrostatic biprism, careful preparation of the sample is required so that it lies adjacent to a region of vacuum, the need to record fine interference fringes limit the obtainable field of view and artefacts result if the biprism is not carefully aligned^32^. Inline holography using through-focus series^33^ reduces the experimental complexity, but research is ongoing to obtain good phase accuracy over a large range of spatial frequencies^27^,^34^; recent work has suggested a hybrid approach that combines the good high spatial frequency performance of inline holography with the accurate low frequency phase imaging of off-axis holography^35^.

Like through-focus inline holography, SAP uses multiple measurements to condition the phase retrieval problem, but in SAP lateral image shifts between each measurement replace longitudinal shifts (variations in defocus). (A recent innovation from the X-ray community combined both kinds of shift^36^.) Inline holography uses a relatively small number of exposures and doesn’t require mechanical movement of the specimen, so it is still relatively quick and is efficient with dose. However, implementing lateral image shifts by translating the specimen, as in SAP, extends the field of view, in principle indefinitely, retains a constant microscope transfer function for every measurement, and has the key advantage of allowing automated, accurate removal of the illumination profile from the reconstructed image, providing freedom to richly structure the ptychographic probe. By so doing, a given feature of a specimen is illuminated by a different composition of spatial frequencies at each specimen position, meaning information about that feature is expressed in different parts of the frequency spectrum of the respective diffraction patterns. This maximises diversity in the recorded data. Although the illumination in the work reported here is approximately flat, the hard edge of the SAA does go some way towards introducing a range of spatial frequencies into the ‘virtual’ probe: many variations on our experimental geometry can be envisaged to draw further value from this concept, for example replacing or supplementing the SAA with a diffuser, as has been shown in X-ray near-field ptychography^26^, or deliberately aberrating the electron beam.

The algorithmic advances we have reported are equally applicable to alternative versions of electron ptychographic CDI, but our SAP experimental implementation has a number of advantages. The probe does not vary over the course of a SAP experiment (a requirement for accurate reconstructions) because no lens or coil settings are changed during data collection; this contrasts with beam-scanning geometries where ensuring a constant probe profile is difficult^15^. Convergent beam electron ptychography relies on a multiplicative model of the interaction of the probe and specimen, the accuracy of which suffers as specimen thickness increases or the required resolution reduces^7^; in SAP the interaction of the SAA with the electron beam can always be modelled reliably by a multiplication. Finally, parallel illumination provides an optimum level of beam coherence^37^ and collection of data for SAP is straightforward and uses a familiar microscope geometry. On the other hand, our method currently takes an average of seven seconds per diffraction pattern recording, making it markedly slower than scanned-probe ptychography, which can also realise higher resolutions. A revived interest in the original point-scanning form of ptychography highlights these benefits and is generating promising results^3^.

That many sources of error in our experiments have been corrected computationally is testament to the general robustness of the ptychographic approach to CDI. This does not imply, however, that improvements to the experiment itself should not be sought. An accurate (piezo) translation stage would reduce the burden on the computational correction of positioning errors and speed up acquisition time. Using different intermediate lens settings to adjust the focal plane of the detector may eliminate optic axis drift. Higher resolutions could be realised by stitching together multiple different exposures of each diffraction pattern to increase dynamic range, or by optimising the magnification of the intermediate lenses. Incorporating energy filtering on a suitably equipped microscope would be straightforward and perhaps essential for thicker and more strongly scattering samples. (It would be fascinating, though, to try adapting our computational filtering to model these situations.)

Accurate, sensitive imaging of the electron phase signal has important applications in the study of the magnetic properties of materials^38^ and in semiconductor characterisation^39^,^40^. Our Future work aims to optimise our experiments, to critically compare performance to that of the two principle kinds of holography, and to apply SAP to the measurement of variations in potential across doped semiconductor junctions.

## Methods

Experiments were carried out on an FEI TF20 200 KeV FEG TEM, with the acquisition program implemented within Gatan Inc.’s Digital Micrograph software suite. A region of interest was first selected from a brightfield survey image. A 10 μm SAA selected a circular area on the specimen 208 nm in diameter, and the microscope’s stepper motor positioning stage was used to translate the specimen in a raster fashion, using a grid of 400 positions (20 rows by 20 columns) with a programmed step size of 62 nm. The stage was over-scanned before the beginning of each row to minimise the effect of backlash. Initial characterisation of the stage movement over the grid was carried out by collecting brightfield images at each position and cross-correlating to measure their offsets. Using this procedure we determined that after the first few rows, the grid was reasonably accurate upon applying scale factors of 0.68 in the *x*-direction and 0.88 in the *y*-direction. We therefore discarded the first few rows of data and corrected any remaining inaccuracies algorithmically, using the method of Maiden *et al.*^24^ (see Supplementary Information).

Diffraction patterns were recorded at a calibrated camera length of 2.95 m (2 m nominal) using spot size 9 and a convergence semi-angle of ~5 mrad. To reduce the dynamic range of the recorded data, the diffraction focus control was used to adjust the image plane of the detector, resulting in recorded diffraction patterns similar to Fig. 2b. The offset of the focal plane from the SAA plane was measured using a geometric argument, leading to equation 1:


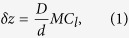


where *M* is the objective lens magnification (48 in our case), *D* is the measured diameter of the SAA, *d* is the measured diameter of the bright disc in the diffraction patterns and *C*_*l*_ is the calibrated camera length. For the results presented here the offset was measured as *δz* = 580 mm, and this figure was used in our forward model of the propagation of the electron wavefront from the image plane to the diffraction plane. The magnification of the intermediate lenses was calculated as:


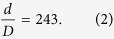


The sample was left to stabilise under the beam for 20 minutes before data collection began, to allow time for the polystyrene spheres to contract slightly. Diffraction patterns were then recorded using an Orius SC200 CCD with an exposure time of 1 second and a binning factor of two. Two exposures per position were captured and averaged to reduce noise. The motorised stage was allowed a settle time of 2 seconds after moving, so that we were able to record diffraction patterns at a rate of one every 7 seconds (See Supplementary Video 1). The central 512 × 512 pixels of each diffraction pattern were used in the reconstruction.

The reconstruction algorithm was coded in MATLAB and used GPU acceleration. Each iteration of the algorithm took ~5 seconds. The shallow phase gradients toward the centres of the polystyrene spheres took a couple of hundred iterations to develop, but the structure of the carbon replica and the higher spatial frequencies at the edges of the spheres were well reconstructed within 10 iterations.

## Additional Information

**How to cite this article**: Maiden, A. M. *et al.* Quantitative electron phase imaging with high sensitivity and an unlimited field of view. *Sci. Rep.* **5**, 14690; doi: 10.1038/srep14690 (2015).

## Supplementary Material

Supplementary Information

Supplementary video 1

Supplementary video 2

Supplementary video 3

## References

[b1] Hoppe W. Trace structure analysis, ptychography, phase tomography. Ultramicroscopy 10, 187–198 (1982).

[b2] Rodenburg J. M. The phase problem, microdiffraction and wavelength-limited resolution—a discussion. Ultramicroscopy 27, 413–422 (1989).

[b3] Pennycook T. J. *et al.* Efficient phase contrast imaging in STEM using a pixelated detector. Part 1: Experimental demonstration at atomic resolution. Ultramicroscopy 151, 160–167 (2015). 25458189 10.1016/j.ultramic.2014.09.013

[b4] Miao J., Ishikawa T., Robinson I. K. & Murnane M. M. Beyond crystallography: Diffractive imaging using coherent x-ray light sources. Science 348, 530–535 (2015). 25931551 10.1126/science.aaa1394

[b5] Rodenburg J. M. & Faulkner H. M. L. A phase retrieval algorithm for shifting illumination. Applied Physics Letters 85, 4795–4797 (2004).

[b6] Guizar-Sicairos M. & Fienup J. R. Phase retrieval with transverse translation diversity: a nonlinear optimization approach. Optics express 16, 7264–7278 (2008). 18545432 10.1364/oe.16.007264

[b7] Thibault P. *et al.* High-resolution scanning x-ray diffraction microscopy. Science 321, 379–382 (2008). 18635796 10.1126/science.1158573

[b8] Maiden A. M. & Rodenburg J. M. An improved ptychographical phase retrieval algorithm for diffractive imaging. Ultramicroscopy 109, 1256–1262 (2009). 19541420 10.1016/j.ultramic.2009.05.012

[b9] Wen Z., Yang C., Liu X. & Marchesini S. Alternating direction methods for classical and ptychographic phase retrieval. Inverse Problems 28, 115010, doi: 10.1088/0266-5611/28/11/115010 (2012).

[b10] Hesse R., Luke D. R., Sabach S. & Tam M. K. Proximal Heterogeneous Block Implicit-Explicit Method and Application to Blind Ptychographic Diffraction Imaging. SIAM Journal on Imaging Sciences 8, 426–457 (2015).

[b11] Humphry M., Kraus B., Hurst A., Maiden A. & Rodenburg J. Ptychographic electron microscopy using high-angle dark-field scattering for sub-nanometre resolution imaging. Nature communications 3, 730, 10.1038/ncomms1733 (2012). PMC3316878 22395621

[b12] D’Alfonso A. J. *et al.* Deterministic electron ptychography at atomic resolution. Physical Review B 89, 064101, 10.1103/PhysRevB.89.064101 (2014).

[b13] McCartney M. R. & Smith D. J. Electron holography: phase imaging with nanometer resolution. Annu. Rev. Mater. Res. 37, 729–767 (2007).

[b14] Hüe F., Rodenburg J., Maiden A., Sweeney F. & Midgley P. Wave-front phase retrieval in transmission electron microscopy via ptychography. Physical Review B 82, 121–415, 10.1103/PhysRevB.82.121415 (2010).

[b15] Hüe F., Rodenburg J., Maiden A. & Midgley P. Extended ptychography in the transmission electron microscope: Possibilities and limitations. Ultramicroscopy 111, 1117–1123 (2011). 21741342 10.1016/j.ultramic.2011.02.005

[b16] Maiden A. M., Humphry M. J. & Rodenburg J. M. Ptychographic transmission microscopy in three dimensions using a multi-slice approach. Journal of the Optical Society of America A 29, 1606–1614 (2012). 10.1364/JOSAA.29.001606 23201876

[b17] Thibault P. & Guizar-Sicairos M. Maximum-likelihood refinement for coherent diffractive imaging. New Journal of Physics 14, 0630–04, doi: 10.1088/1367-2630/14/6/063004 (2012).

[b18] Edo T. *et al.* Sampling in x-ray ptychography. Physical Review A 87, 053850, 10.1103/PhysRevA.87.053850 (2013).

[b19] Thibault P. & Menzel A. Reconstructing state mixtures from diffraction measurements. Nature 494, 68–71 (2013). 23389541 10.1038/nature11806

[b20] Batey D. J., Claus D. & Rodenburg J. M. Information multiplexing in ptychography. Ultramicroscopy 138, 13–21 (2014). 24413077 10.1016/j.ultramic.2013.12.003

[b21] Clark J. N., Huang X., Harder R. J. & Robinson I. K. Dynamic Imaging Using Ptychography. Physical Review Letters 112, 113901, 10.1103/PhysRevLett.112.113901 (2014). 24702370

[b22] Deng J. *et al.* Continuous motion scan ptychography: characterization for increased speed in coherent x-ray imaging. Optics Express 23, 5438–5451 (2015). 25836777 10.1364/OE.23.005438 PMC4394751

[b23] Tripathi A., McNulty I. & Shpyrko O. G. Ptychographic overlap constraint errors and the limits of their numerical recovery using conjugate gradient descent methods. Optics Express 22, 1452–1466 (2014). 24515152 10.1364/OE.22.001452

[b24] Maiden A., Humphry M., Sarahan M., Kraus B & Rodenburg J. An annealing algorithm to correct positioning errors in ptychography. Ultramicroscopy 120, 64–72 (2012). 22813888 10.1016/j.ultramic.2012.06.001

[b25] Zhang F. *et al.* Translation position determination in ptychographic coherent diffraction imaging. Optics Express 21, 13592–13606 (2013). 23736612 10.1364/OE.21.013592

[b26] Stockmar M. *et al.* Near-field ptychography: phase retrieval for inline holography using a structured illumination. Sci. Rep. 3, 1927, 10.1038/srep01927 (2013). 23722622 PMC3668322

[b27] Koch C. T. Towards full-resolution inline electron holography. Micron 63, 69–75 (2014). 24239416 10.1016/j.micron.2013.10.009

[b28] Burdet N. *et al.* Evaluation of partial coherence correction in X-ray ptychography. Optics Express 23, 5452–5467 (2015). 25836778 10.1364/OE.23.005452

[b29] Wang Y. C., Chou T. M., Libera M., Voelkl E. & Frost B. G. Measurement of Polystyrene Mean Inner Potential by Transmission Electron Holography of Latex Spheres. Microscopy and Microanalysis 4, 146–157 (1998).

[b30] Chou T. M. & Libera M. Mean free paths for inelastic electron scattering in silicon and poly(styrene) nanospheres. Ultramicroscopy 94, 31–35 (2003). 12489593 10.1016/s0304-3991(02)00192-4

[b31] Lichte H. & Lehmann M. Electron holography—basics and applications. Reports on Progress in Physics 71, 016102, doi: 10.1088/0034-4885/71/1/016102 (2008).

[b32] Lichte H. *et al.* Artefacts in electron holography. Ultramicroscopy 64, 67–77 (1996).

[b33] Koch C. T. A flux-preserving non-linear inline holography reconstruction algorithm for partially coherent electrons. Ultramicroscopy 108, 141–150 (2008). 17485172 10.1016/j.ultramic.2007.03.007

[b34] Haigh S. J., Jiang B., Alloyeau D., Kisielowski C. & Kirkland A. I. Recording low and high spatial frequencies in exit wave reconstructions. Ultramicroscopy 133, 26–34 (2013). 23751209 10.1016/j.ultramic.2013.04.012

[b35] Ozsoy-Keskinbora C., Boothroyd C., Dunin-Borkowski R., van Aken P. & Koch C. Hybridization approach to in-line and off-axis (electron) holography for superior resolution and phase sensitivity. Scientific reports 4, 7020, 10.1038/srep07020 (2014). 25387480 PMC4228327

[b36] Robisch A. L., Kröger K., Rack A. & Salditt, T. Near-field ptychography using lateral and longitudinal shifts. New Journal of Physics 17, 073033, doi: 10.1088/1367-2630/17/7/073033 (2015).

[b37] Morishita S., Yamasaki J. & Tanaka N. Measurement of spatial coherence of electron beams by using a small selected-area aperture. Ultramicroscopy 129, 10–17 (2013). 23545433 10.1016/j.ultramic.2013.02.019

[b38] Pozzi G., Beleggia M., Kasama T. & Dunin-Borkowski R. E. Interferometric methods for mapping static electric and magnetic fields. Comptes Rendus Physique 15, 126–139 (2014).

[b39] Twitchett A., Dunin-Borkowski R. & Midgley P. Quantitative electron holography of biased semiconductor devices. Physical review letters 88, 238302, 10.1103/PhysRevLett.88.238302 (2002). 12059403

[b40] Hytch M., Houdellier F., Hue F. & Snoeck E. Nanoscale holographic interferometry for strain measurements in electronic devices. Nature 453, 1086–1089 (2008). 18563161 10.1038/nature07049

