# Supplementary material for: Quantitative electron phase imaging with high sensitivity and an unlimited field of view
Source: Sci Rep. 2015 Oct 1;5:14690. doi: 10.1038/srep14690 (PMC4589788; doi:10.1038/srep14690)
Supplement: Supplementary Information [file srep14690-s1.pdf]

Quantitative electron phase imaging with high sensitivity and an unlimited field of view:

Supplementary Information

A. M. Maiden[a,*], M. C. Sarahan[c], M. D. Stagg[b,c], S. M. Schramm[b] and M. J. Humphry[b],

[a.] Dept. Electronic & Electrical Engineering, University of Sheffield, Mappin St, Sheffield, S1 3JD

[b.] Phase Focus Ltd, Electric Works, Sheffield Digital Campus, Sheffield S1 2BJ

[c.] Gatan Inc, 5794 W. Las Positas Blvd, Pleasanton, CA, 94588

[*] Corresponding Author –

Email: a.maiden@sheffield.ac.uk,

Tel: +44 114 2225181

Address: Dept. Electronic & Electrical Engineering,

University of Sheffield,

Mappin St,

Sheffield,

S1 3JD, UK

Details of the reconstruction procedure

Our reconstruction algorithm follows the format of the ePIE technique[1], where diffraction patterns are addressed sequentially. A parallel-update alternative would be straightforward to implement by replacing the conventional Fourier update step in existing parallel algorithms[2] with the version described below.

Data consists of a set of $j = 1 \dots$ J measured diffraction patterns $I_j(\mathbf{u})$, where $\mathbf{u} = (u, v)$ is a coordinate in the detector plane. Each diffraction pattern was recorded at a different specimen position, with the measurement of this position corresponding to the j^{th}diffraction pattern denoted $\mathbf{R}_j = (R_{x,j}, R_{y,j})$.

Reconstruction begins with initial estimates of the shape of the selected area aperture (SAA) and of the specimen transmission function. In keeping with the rest of the ptychography literature, we will denote the specimen estimate the 'object', $O(\mathbf{r})$, even though the object in our case is actually the electron wavefront incident at the plane of the SAA. Similarly the estimate of the SAA (the ptychographic 'probe' in this case) we denote $P(\mathbf{r})$. $\mathbf{r} = (x, y)$ is a coordinate in the plane of the SAA. We use a circular aperture of approximately the correct diameter as an initial model of the SAA and free space as an initial estimate of the specimen. An initial estimate of the diffuse background intensity, $B(\mathbf{u})$, present in each diffraction pattern is also required – a constant value of $1/N$ for every pixel suffices, where N is the number of pixels per diffraction pattern ($N=512^2$ in our results).

A single sub-iteration of the algorithm uses one randomly chosen diffraction patterns to update the current object and probe estimates, following the steps shown in Figure S1. Each sub-iteration also updates the following quantities: a correction vector, $\mathbf{C}_j = (C_{x,j}, C_{y,j})$, for the jth specimen position; an optic axis drift vector, $\mathbf{D}_j = (D_{x,j}, D_{y,j})$; the width of the

convolution kernel, σ (used to model the partial spatial coherence of the electron wave), and a weighting, w_j, to apply to $B(\mathbf{u})$. All of these parameters are initialised to zero. A full iteration is completed by carrying out one sub-iteration for every diffraction pattern and then updating $B(\mathbf{u})$.

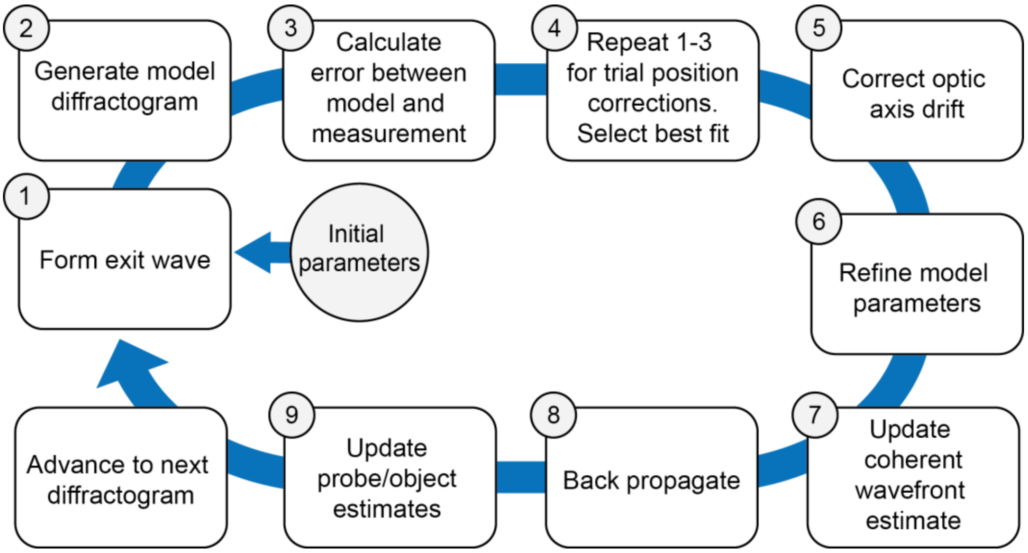

Figure S1: **An overview of the reconstruction process.** One sub-iteration of the algorithm follows the loop to update current estimates of the object, probe and various parameters of the diffraction pattern model. A full iteration is completed by carrying out a sub-iteration for every recorded diffraction pattern.

The steps in the algorithm are described below, with reference to the numbering in Figure S1.

Step 1: An exit wave is generated by multiplying current estimates of the SAA and the specimen image with appropriate shift applied:

$$\psi_j(\mathbf{r}) \ = O(\mathbf{r} - \mathbf{R}_j - \mathbf{C}_j)P(\mathbf{r}), \tag{S1}$$

Step 2: $\psi_j(\mathbf{r})$ is propagated over the distance δz from the plane of the SAA to the plane imaged by the detector. The Fresnel propagator $\mathcal{P}_z$ is used:

$$\mathcal{P}_z[f(\mathbf{r})] = \ \mathcal{F}^{-1}[\mathcal{F}[f(\mathbf{r})]\exp(-\mathrm{j}\pi\lambda z\mathbf{q}^2)], \tag{S2}$$

Where $\mathcal{F}$ represents a Fourier transform with reciprocal coordinate $\mathbf{q}$ and $\lambda = 2.51\text{pm}$ is the wavelength of the electron beam at 200keV.

The resulting wavefront at the plane imaged by the detector is:

$$\Psi_j(\mathbf{u}) = \mathcal{P}_{\delta z}\left[\psi_j(\mathbf{r})\right], \tag{S3}$$

with $\delta z = 580\text{mm}$ for our results.

Using current estimates of the Gaussian convolution kernel width, σ, the background intensity, $B(\mathbf{u})$, and the background weighting, w_j, the diffraction pattern intensity is modelled as:

$$M(\mathbf{u}) = \left|\Psi_j(\mathbf{u})\right|^2 \otimes G(\mathbf{u}, \sigma) + w_j B(\mathbf{u}), \tag{S4}$$

where $\otimes$ indicates a convolution, implemented via multiplication in the Fourier domain. The Fourier transform of the Gaussian kernel $G(\mathbf{u}, \sigma)$ is:

$$\tilde{G}\left(\mathbf{q}, \sigma_j\right) = \mathcal{F}[G(\mathbf{u}, \sigma)] = \delta + (1 - \delta)\exp(-2\pi^2\sigma^2|\mathbf{q}|^2). \tag{S5}$$

The small constant δ (equal to 0.05 for our results) prevents instabilities caused by zero-divisions in step 7. The kernel is normalised so that different values of σ can be fairly compared in step 6.

Step 3: The model of equation S4 is compared to the recorded diffraction pattern. Because of the drift of the optic axis, the modelled and recorded intensities may be offset from each other, so we use a shift-invariant error metric:

$$E_{pos} = \frac{\sum_{\mathbf{v}}\left(\left|\tilde{M}(\mathbf{q})\right| - \left|\tilde{I}_J(\mathbf{q})\right|\right)^2.}{\sum_{\mathbf{v}}\left|\tilde{I}_J(\mathbf{q})\right|^2.} \tag{S6}$$

Here $\widetilde{\mathrm{M}}(\mathbf{q}) = \mathcal{F}[\mathrm{M}(\mathbf{u})]$ is the Fourier transform of the modelled intensity and the Fourier transform of the recorded intensity is $\widetilde{\mathrm{I}}_{\mathrm{j}}(\mathbf{q}) = \mathcal{F}[\mathrm{I}_{\mathrm{j}}(\mathbf{u})]$.

Step 4: Following the procedure of Maiden *et al.*[3], steps 2 and 3 are repeated for several randomly selected perturbations to the current position correction vector $\mathbf{C}_{\mathrm{j}}$. The $\mathbf{C}_{\mathrm{j}}$ and $\Psi_j(\mathbf{u})$ that give the smallest value of E_{pos} are retained for subsequent steps and the next iteration.

Step 5: The recorded intensity, $\mathrm{I}_{\mathrm{j}}(\mathbf{u})$, is cross-correlated with the modelled intensity, $M(\mathbf{u})$. The location of the maximum peak in the cross-correlation, corresponding to the offset of the optic axis from the centre of the detector, is stored as $\mathbf{D}_{\mathrm{j}}$. (We hope to eliminate optic axis drift using improved experimental procedures in future work, enabling us to forego this computationally intensive step.)

Step 6: In a scheme similar to that used to correct position errors, a small number of perturbations to the current value of the Gaussian kernel width are trialled and the result that gives the best match to the recorded data retained for subsequent iterations. Breaking this process into sub-steps:

6a. A trial perturbation, Δ, is applied to the kernel width, σ, in our model to give an estimate, $M_{\mathrm{p.c}}(\mathbf{u})$, of the partially-coherent diffraction pattern:

$$M_{\mathrm{p.c}}(\mathbf{u}) = \left|\Psi_j(\mathbf{u})\right|^2 \otimes G(\mathbf{u}, \sigma + \Delta). \tag{S7}$$

6b. The diffuse background is added to the model:

$$M'(\mathbf{u}) = M_{\mathrm{p.c}}(\mathbf{u}) + w_j B(\mathbf{u}). \tag{S8}$$

Where w_j is calculated to minimise the quantity:

$$E_{model} = \frac{\sum_{\boldsymbol{u}} \left(I_j(\mathbf{u} - \mathbf{D}_\mathrm{j}) - M'(\mathbf{u}) \right)^2}{\sum_{\boldsymbol{u}} I_j(\mathbf{u})^2} \tag{S9}$$

6c. Steps 6a and 6b repeat for a small number of trial perturbations.

6d. The adjustments to w_j and σ that resulted in the smallest value of E_{model} are retained. Using the $M_{\mathrm{p.c}}(\mathbf{u})$ generated from these current-best values, the residue,

$S_j(\mathbf{u}) = I_j(\mathbf{u} - \mathbf{D}_\mathrm{j}) - M_{\mathrm{p.c}}(\mathbf{u})$, is calculated and stored for use later in the update of $B(\mathbf{u})$.

Step 7: $\Psi_\mathrm{j}(\mathrm{u})$ is updated in the manner of Clark & Peele[4]:

$$\Psi'_j(\mathbf{u}) = \Psi_j(\mathbf{u}) \frac{I_j(\mathbf{u}\text{-}\mathbf{D}_\mathrm{j})}{M'(\mathbf{u})} \tag{S10}$$

Step 8: The revised wavefront is propagated back to the plane of the SAA:

$$\psi'_j(\mathbf{r}) = \mathcal{P}_{-\delta z}\left[\Psi'_j(\mathbf{u})\right] \tag{S11}$$

Step 9: New estimates of the specimen and probe are formed using the ePIE update rules:

$$\begin{aligned} \mathrm{O}'(\mathrm{r}) &= \mathrm{O}(\mathrm{r}) + \alpha \frac{\mathrm{P}^*(\mathbf{r} + \mathbf{R}_\mathrm{j} + \mathbf{C}_\mathrm{j})}{|\mathrm{P}(\mathbf{r})|^2_{\mathrm{max}}} \left(\psi'_\mathrm{j}(\mathbf{r}) - \psi_\mathrm{j}(\mathbf{r}) \right) \\ \mathrm{P}'(\mathbf{r}) &= \mathrm{P}(\mathbf{r}) + \beta \frac{\mathrm{O}^*(\mathbf{r} - \mathbf{R}_\mathrm{j} - \mathbf{C}_\mathrm{j})}{|\mathrm{O}(\mathbf{r})|^2_{\mathrm{max}}} \left(\psi'_\mathrm{j}(\mathbf{r}) - \psi_\mathrm{j}(\mathbf{r}) \right) \end{aligned} \tag{S12}$$

α and β control the update rate of the object and probe; values of $\alpha = 1, \beta = 0.5$ were used for our results.

Steps 1-9 are repeated for each diffraction pattern. To complete an iteration of the reconstruction $B(\mathbf{u})$ is updated:

$$\mathrm{B}(\mathbf{u}) = \mathrm{B}(\mathbf{u}) + \gamma\left(\frac{\sum_{\mathrm{j}} \mathrm{S_j}(\mathbf{u})}{\sum_{\mathbf{u}}\sum_{\mathrm{j}} \mathrm{S_j}(\mathbf{u})} - \mathrm{B}(\mathbf{u})\right) \tag{S13}$$

Here γ is a constant (0.25 for the results presented) that controls the rate of update.

Additional details of the results

To obtain the results shown in the main article, the algorithm detailed above was run twice. In the first run, consisting of 500 iterations, partial coherence effects were not included in the diffraction pattern model and the amplitude of the specimen image was restricted to the range 0.2-1. Phase vortices were then removed from the reconstructed specimen image, following Stockmar *et al.*[5]. The position corrections, background intensity, probe estimate and the phase part of the vortex-corrected specimen estimate were used to seed the second run, over 100 iterations, which included partial coherence. This two-stage approach helped limit the number of phase vortices within the images of the polystyrene spheres, a common artefact for this kind of specimen[5]. The vortex-removal step was not needed for the reconstruction shown in Figure 4, which did not contain any vortices after the first run of the algorithm.

The various plots of Figures S2, S3, S4 and S5, described below, provide further details of the result shown in **Figure 2** of the main article.

Accuracy of the diffraction pattern model

To arrive at our model, we assumed the microscope formed a partially spatially coherent elastic image superposed with an inelastic background at the plane of the SAA.

We assumed a diffuse incoherent background that when propagated to the plane imaged by the detector was approximately the same for every specimen position, varying only in intensity depending on the mean thickness of the area of the specimen selected by the SAA.

The form of the background, $B(\mathbf{u})$, and the value of the intensity weightings, w_j, were derived iteratively as described above, giving results that we will come to shortly.

We further assumed a Gaussian form to the spatial coherence envelope that reduced fringe contrast in our recorded data. During each sub-iteration of the second run through the algorithm, the current value of the width of the kernel that defines this envelope, σ, was compared against two random perturbations. Initially these perturbations were selected from within bounds of ±5nm, reducing linearly to zero over 75 iterations. The value of σ converged within 10-20 iterations to a steady value of 120nm.

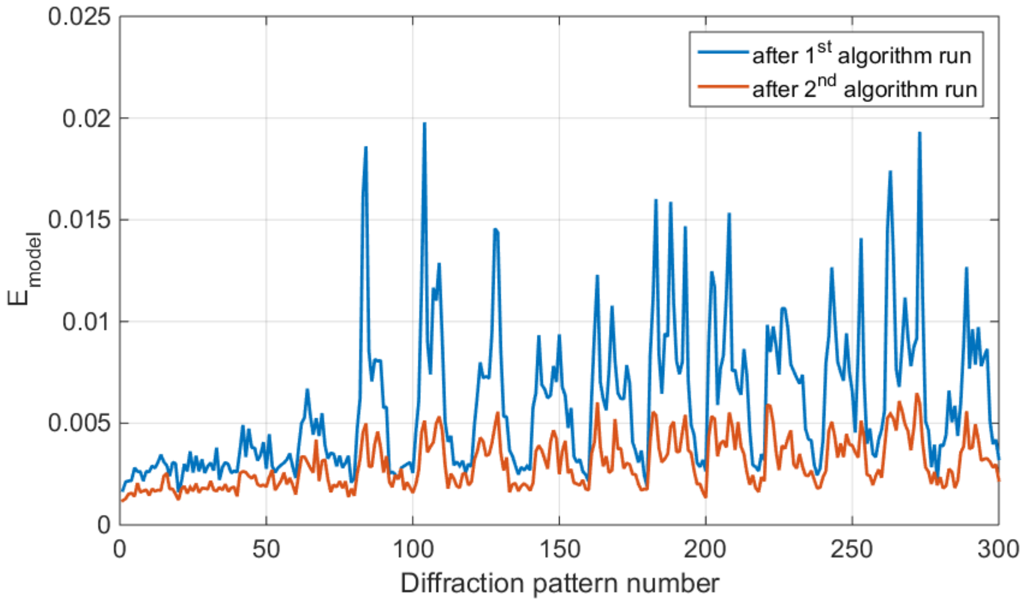

Figure S2: **The accuracy of the diffraction pattern model.** The error between the modelled and the measured diffraction patterns falls by around 50% for the second run of the algorithm (which includes the effects of partial spatial coherence) compared to the first run (which assumed full coherence). Peaks in the error correspond to specimen positions where polystyrene spheres are under the SAA.

Figure S2 plots the value of E_{model} (Equation S9) as a function of diffraction pattern number. The error after the second run of the algorithm, which accounted for partial spatial coherence, drops to around half that of the first run where perfect coherence was assumed. The

systematic peaks in the error occur when the polystyrene spheres fall under the SAA, which we think is due to our assumption of a constant form for $B(\mathbf{u})$.

We are currently investigating several refinements to our model. One idea is to allow for variation in the diffuse background between the different specimen positions, another is to model partial spatial coherence using modes[6], which would remove the constraint of a Gaussian form to the coherence kernel.

Position correction

The initial specimen positions, $\mathbf{R}_j$, provided to the algorithm were derived by scaling the positions read back from the translation stage by factors derived from calibration tests, which indicated that the obtained positions agreed with the read back positions after application of a 0.88 scaling factor in the y-axis (standard deviation: 0.007) and a 0.68 scaling factor in the x-axis (standard deviation: 0.02). The first five rows of diffraction patterns were discarded from the data supplied to the algorithm as positioning was unreliable for a short duration after the initial large stage movement to the first position. The remaining 300 positions are plotted as circles in Figure S3.

Beginning after ten iterations of the reconstruction, the running estimates of each $\mathbf{C}_\mathrm{j}$ were compared against two random perturbations, with the correction vectors that gave the best fit to the recorded diffraction patterns becoming the $\mathbf{C}_\mathrm{j}$ for the following iteration. As described in greater detail by Maiden *et al.*[3], an annealing approach was taken whereby these random perturbation vectors were chosen from within a slowly decreasing radius. For our results this radius was set initially to 20 pixels and fell to zero linearly over 450 iterations of the first run of the algorithm. The final correction vectors are shown in Figure S3 as lines originating at their corresponding initial positions. The mean positioning error corrected was 7.7 pixels

(9.8nm) and the maximum correction was 32.3 pixels (41nm), or 20% of the diameter of the virtual probe formed by the SAA.

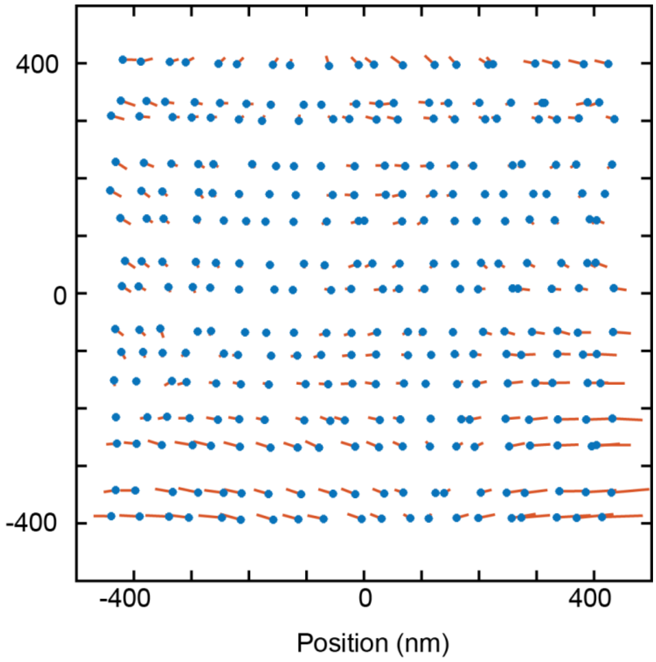

Figure S3: **The correction of positioning errors.** The calibrated specimen positions (blue dots) were obtained by scaling the positions read back from the translation stage using scale factors determined during initial testing. These positions were refined by an algorithmic procedure, resulting in the correction vectors shown as orange lines in the Figure.

Drift correction

The final set of optic axis offsets, $\mathbf{D}_j$, are shown in Figure S4. To reduce computation time, the cross-correlation step was restricted to single pixel precision. The action of the drift correction can also be observed by comparing Supplementary Video 1, showing the raw data, with Supplementary Video 2, in which the optic axis has been recentred using the vectors plotted in Figure S4.

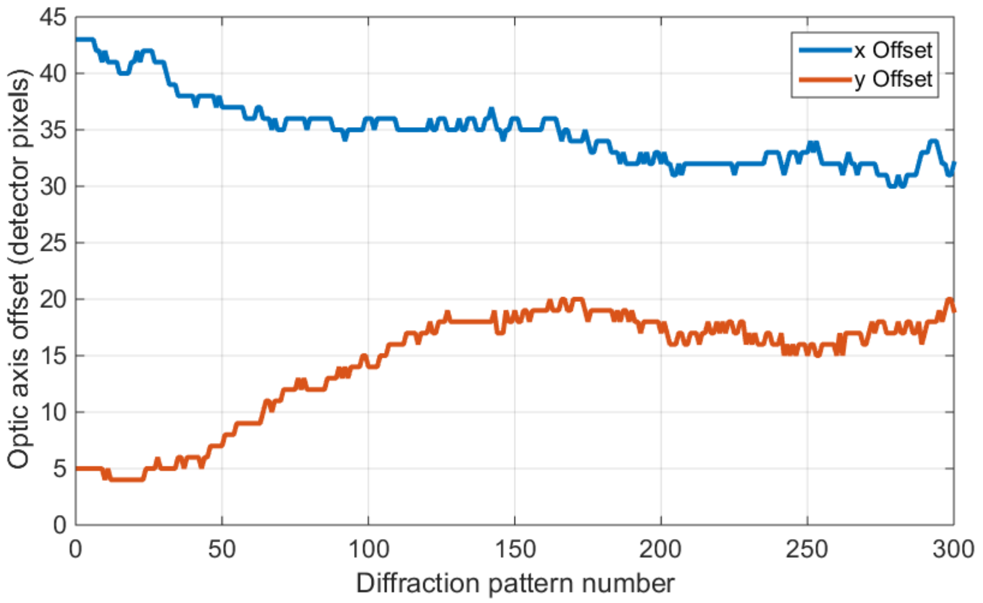

Figure S4: The correction of optic axis drift. The centre point of the optic axis relative to the centre of the detector was estimated during each sub-iteration of the reconstruction algorithm by cross-correlating the modelled and the measured diffraction patterns. The final offsets recovered for each diffraction pattern are shown – spikes in the plots are the result of cross-correlating to single-pixel precision.

Recovered diffuse background and background weightings

Although using the SAA to mask the electron beam downstream of the specimen is not dose efficient, it does allow us to treat the inelastic background present in the image in a coarsely localised fashion, so that we can compensate for it differently depending on the local thickness of the specimen – this is the task assigned to the background function, $B(\mathbf{u})$, and the weighting function, w_j, whose final forms are shown in Figure S5.

Figure S5a shows the recovered diffuse background signal $B(\mathbf{u})$, scaled by the average value of w_j to indicate roughly the number of counts that are lost to this background (for reference, the diffraction patterns had a mean maximum pixel value of ~10000 counts). Figure S5b and S5c provide two representations of the final weightings, w_j, applied to $B(\mathbf{u})$ at each specimen position. In Figure S5b, w_j is plotted as a function of diffraction pattern number. Here the weighting has been scaled to a proportion of the total recorded counts in each diffraction pattern, so a value of 0.2 indicates that the source of 20% of the total counts recorded in the

corresponding diffraction pattern resulted from the incoherent background. The peaks in Figure S5b correspond, as expected, with the location of the polystyrene spheres in the image, where inelastic scatter is more prominent. To visualise this, Figure S5c plots w_j as a function of specimen position. To generate this map the recovered image of the SAA was used as a mask to select a region of pixels corresponding to specimen position 1, then the value of w_1 was added to these pixels. This process was repeated for each specimen position, adding w_2 to the pixels masked at specimen position 2 and so on. The value of each pixel was then divided by the number of times it was selected by the mask to give a coarse representation of the amount of diffuse inelastic scatter across the specimen.

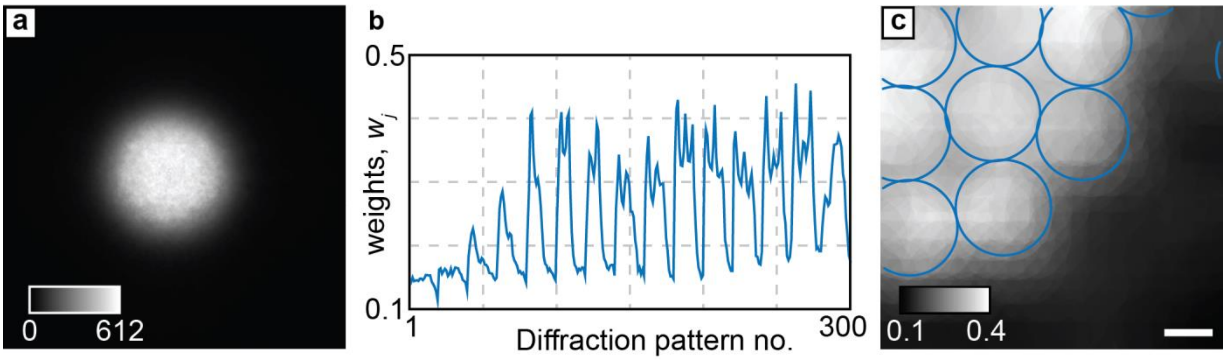

Figure S5: Details of the model parameters recovered during image reconstruction. a, The diffuse background, which contributed on average around 25% of the recorded counts to each diffraction pattern. **b**, The weighting of the diffuse background at each specimen position, plotted as a proportion of the total recorded counts. The weighting increases in positions where the polystyrene spheres fall under the SAA due to an increase in inelastic scatter, as **c**, the map of the weights as a function of position shows. The circles in **c** indicate the positions of the polystyrene spheres. Scale bars 100nm.

Explanation of the fitting process

To estimate polystyrene's mean inner potential, v_0, and inelastic mean free path, λ, from our reconstructions, we used a similar process to that detailed by Latychevskaia *et al.*[7].

First, radial averages for the polystyrene spheres were generated using manually selected centre points and radii. To avoid using data from near the centre of the spheres, where the

radial average consisted of only a few pixels and so was distorted by the underlying carbon substrate, and at the edges, where the profiles were distorted in regions where the spheres meet, fitting was performed using data between 20-80% of the spheres' radii.

The spheres were assumed to be perfectly spherical, so that their thickness along the optic axis at a distance *r* (in pixels) from their centres could be calculated as:

$$t(r) = 2\sqrt{R^2 - (r.\mathrm{dx})^2}, \tag{S14}$$

where R is the sphere radius and $\mathrm{dx} = 1.27$ nm is the pixel pitch in the reconstructed phase image.

To calculate a value for v_0 a least squares fit was carried out to find the constants *a* and *b* that minimised the quantity:

$$E = \sum_{r=0.2R}^{0.8R} (t(r) - a\theta(r) - b)^2, \tag{S15}$$

where $\theta(r)$ represents the radial average of the unwrapped phase of each sphere. The value of *a* relates to v_0 according to:

$$v_0 = {}^{1}/_{aE_C}, \tag{S16}$$

where E_c is an energy-dependent constant ($= 7.288 \times 10^{-3}$ rad/V/nm at 200keV).

To calculate a value for λ the same fitting routine was used to find constants *c* and *d* to minimise the quantity:

$$E = \sum_{r=0.2R}^{0.8R} (t(r) - c\ln(A(r)) - d)^2, \tag{S17}$$

where $A(r)$ represents the radial average of the reconstructed amplitude of each polystyrene sphere The value of *c* relates to λ according to:

$$\lambda = -{}^{c}/_{2}. \quad \text{(S18)}$$

The process above was carried out for each of the 8 full spheres shown in Figure 2 of the main article, and the 4 complete spheres shown in Figure 4a. The mean of these measurements gave our final estimates of the two quantities.

References

1 Maiden, A. M. & Rodenburg, J. M. An improved ptychographical phase retrieval algorithm for diffractive imaging. *Ultramicroscopy* **109**, 1256-1262 (2009).

2 Thibault, P. *et al.* High-resolution scanning x-ray diffraction microscopy. *Science* **321**, 379-382 (2008).

3 Maiden, A., Humphry, M., Sarahan, M., Kraus, B. & Rodenburg, J. An annealing algorithm to correct positioning errors in ptychography. *Ultramicroscopy* **120**, 64-72 (2012).

4 Clark, J. N. & Peele, A. G. Simultaneous sample and spatial coherence characterisation using diffractive imaging. *Applied Physics Letters* **99**, 154103 (2011).

5 Stockmar, M. *et al.* X-Ray Near-Field Ptychography for Optically Thick Specimens. *Physical Review Applied* **3**, 014005 (2015).

6 Thibault, P. & Menzel, A. Reconstructing state mixtures from diffraction measurements. *Nature* **494**, 68-71 (2013).

7 Latychevskaia, T., Formanek, P., Koch, C. T. & Lubk, A. Off-axis and inline electron holography: Experimental comparison. *Ultramicroscopy* **110**, 472-482 (2010).
